# Supplementary material for: Revealing concealed cardioprotection by platelet Mfsd2b-released S1P in human and murine myocardial infarction
Source: Nat Commun. 2023 Apr 26;14:2404. doi: 10.1038/s41467-023-38069-5 (PMC10133218; doi:10.1038/s41467-023-38069-5)
Supplement: Supplementary file 1 — Supplementary Information [file 41467_2023_38069_MOESM1_ESM.pdf]

Revealing concealed cardioprotection by platelet Mfsd2b-released S1P in human and murine myocardial infarction

Supplementary Table 1: Functional echocardiography data over time

Group	n	time after AMI	heart rate [bpm]	stroke volume [µl]	cardiac output [ml min^{-1}]	ejection fraction [%]
sham	6	Baseline	438.6 ± 23.3	36.21 ± 7.63	15.84 ± 3.16	53.98 ± 3.12
NaCl	7		482.4 ± 46.1	33.01 ± 3.47	15.13 ± 2.00	51.41 ± 6.63
SNT -	6		459.9 ± 48.7	32.42 ± 4.80	14.91 ± 2.66	50.99 ± 5.57
SNT +	7		448.7 ± 46.2	34.65 ± 4.24	15.47 ± 2.64	50.91 ± 3.95
sham	6	24h post	510.3 ± 42.2	30.97 ± 6.85	15.82 ± 3.89	53.77 ± 2.00
NaCl	7		560.3 ± 64.6	19.32 ± 2.84	10.41 ± 2.31	33.43 ± 5.88
SNT -	6		535.8 ± 75.3	19.30 ± 4.07	10.26 ± 2.28	31.01 ± 5.21
SNT +	7		523.9 ± 71.2	23.10 ± 3.86	12.12 ± 2.31	37.67 ± 4.30
sham	6	21d post	463.2 ± 38.0	33.06 ± 2.89	15.30 ± 1.70	52.67 ± 3.24
NaCl	6		501.1 ± 24.6	23.09 ± 2.70	11.56 ± 1.35	38.45 ± 5.23
SNT -	6		508.0 ± 77.3	24.33 ± 2.02	12.33 ± 1.75	39.52 ± 7.63
SNT +	7		508.9 ± 45.1	28.70 ± 4.00	15.53 ± 1.79	46.85 ± 4.54

Additional data to Figure 1g-j in the main text. Four different groups were analyzed: sham-operated, NaCl 0.9%-treated, treated with non-activated platelet supernatant (SNT-), treated with activated platelet supernatant (SNT+). Data are given in mean ± s.d.

Supplementary Table 2: Flow chart of patients who were not eligible for cardiac magnetic resonance imaging

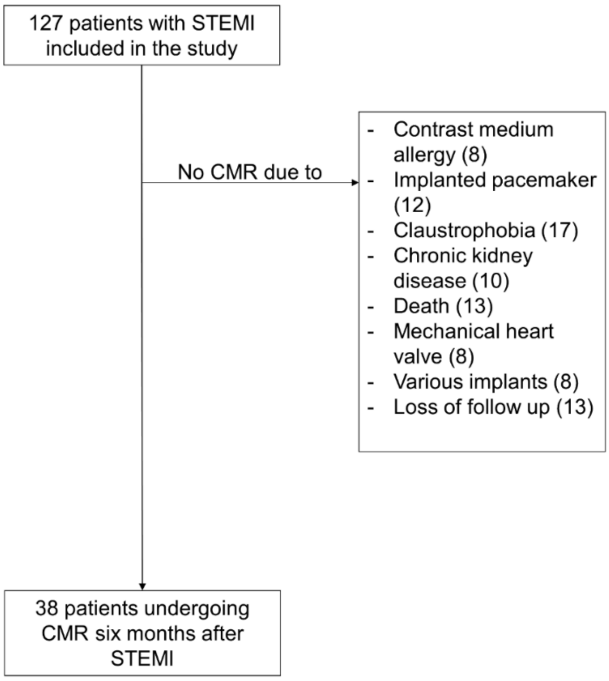

Supplementary Table 3: Patients´ characteristics

Baseline Characteristics	**<1,556 pmol/ml S1P N=42**	**1,556-2,072 pmol/ml S1P N=43**	**2,072 pmol/ml S1P N=42**	**P-Value**
Gender – female/male (%/%)	9/33 (21.4/78.6)	12/31 (27.9/72.1)	13/29 (31.0/69.0)	.608
Age - mean±SD	63.76±13.7	62.28±13.6	62.76±13,5	.878
Weight - mean±SD	84.36±15.1	86.42±14.4	80.03±16.9	.179
BMI -mean±SD	27.48±4.5	28.71±4.1	25.93±4.3	.021
Obesity - no. (%)	7 (16.7%)	11 (27.5%)	6 (15.0%)	.310
Nicotine abuse - no. (%)	12 (29.3%)	20 (46.5%)	14 (34.1%)	.238
Pos. familly history- no. (%)	5 (12.2%)	6 (14.0%)	3 (7.3%)	.610
Diabetes - no. (%)	9 (22.0%)	9 (20.9%)	11 (26.2%)	.831
Prior MI - no. (%)	3 (7.3%)	2 (4.7%)	4 (9.8%)	.664
Prior TIA - no. (%)	0 (0.0%)	2 (4.7%)	0 (0.0%)	.144
Prior Stroke - no. (%)	3 (7.5%)	2 (4.7%)	4 (9.8%)	.664
Prior PCI - no. (%)	4 (9.8%)	4 (9.3%)	9 (21.4%)	.182
Prior CABG - no. (%)	1 (2.4%)	0 (0.0%)	0 (0.0%)	.356
COPD - no. (%)	2 (4.9%)	3 (7.0%)	1 (2.5%)	.637
Pulmonary hypertension- no. (%)	8 (19.0%)	5 (11.6%)	5 (11.9%)	.639
Hypertension - no. (%)	27 (65.9%)	32 (74.4%)	29 (69.0%)	.687
Peripheral arterial occlusive disease - no. (%)	1 (2.4%)	1 (2.3%)	1 (2.4%)	.999
Atrial fibrilation - no. (%)	10 (24.4%)	4 (9.3%)	7 (17.1%)	.181
HLP - no. (%)	11 (26.8%)	15 (35.7%)	11 (26.8%)	.592
Reduction of the left ventricular ejection fraction - no.(%)				.383
None	16 (38.1%)	17(39.5%)	10 (25%)	
Minor	15 (35.7%)	17 (39.5%)	14 (35%)	
Medium	5 (12.0%)	3 (7.0%)	12 (30%)	
High	6 (14.3%)	6 (14.0%)	4 (10%)	

Patients´ characteristics showed no differences between the cohorts. BMI = body mass index, CABG = coronary artery bypass graft, COPD = chronic obstructive pulmonary disease, HLP = hyperlipoproteinemia, MI = myocardial infarction, PCI = percutaneous coronary intervention, TIA = transient ischemic attack

Supplementary Table 4: Patients' co-medication and S1P plasma concentrations

Co-Medication- no(%)	**<1,556 pmol/ml S1P N=42**	**1,556-2,072 pmol/ml S1P N=43**	**2,072 pmol/ml S1P N=42**	**P-Value**
Aspirin	41 (97.6%)	41 (95.3%)	39 (92.9%)	.652
Pre- MI Aspirin	7 (16.7%)	7 (16.3%)	5 (11.9%)	.965
Oral anticoagulation	8 (19.0%)	5 (11.6%)	5 (11.9%)	.822
Marcumar	5 (11.9%)	2 (4.7%)	1 (2.4%)	.431
Tirofiban	10 (23.8%)	9 (20.9%)	8 (19.0%)	.866
L- Thyroxin	4 (9.5%)	4 (9.3%)	7 (16.7%)	.756
ACE inhibitors	31 (73.8%)	34 (79.1%)	25 (59.5%)	.312
Angiotensin Ⅱ receptor antagonists	8 (19.0%)	3 (7.0%)	11 (26.2%)	.140
Beta Blocker	40 (95.2%)	37 (86.0%)	36 (85.7%)	.622
Calcium antagonist	9 (21.4%)	7 (16.3%)	9 (21.4%)	.932
Diuretics	15 (35.7%)	12 (27.9%)	21 (50.0%)	.273
Aldosterone antagonist	4 (9.5%)	4 (9.3%)	6 (14.3%)	.890
Proton pump inhibitor	35 (83.3%)	35 (81.4%)	29 (69.0%)	.509
Statin	36 (85.7%)	40 (93.0%)	39 (92.9%)	.267
Oral antidiabetics	3 (7.1%)	8 (18.6%)	7 (16.7%)	.621
Insulin	2 (4.8%)	3 (7.0%)	5 (11.9%)	.730
Dipyrone	4 (9.5%)	1 (2.3%)	2 (4.8%)	.644
Ibuprofen	1 (2.4%)	2 (4.7%)	2 (4.8%)	.818
Paracetamol	0 (0.0%)	1 (2.3%)	0 (0.0%)	.665
Opiate	0 (0.0%)	2 (4.7%)	2 (4.8%)	.642
Antiarrhythmics	1 (2.4%)	0 (0.0%)	0 (0.0%)	.661
Inhaled Beta-2-agonists	6 (14.3%)	6 (14.0%)	3 (7.1%)	.797
Inhaled glucocorticoids	0 (0.0%)	1 (2.3%)	0 (0.0%)	.665

Patients' co-medication revealed no differences between the three groups. ACE = Angiotensin-converting-enzyme, MI = myocardial infarction

Supplementary Table 5: Multivariable regression of infarct size and patients` characteristics before and after IPTW

	**Unstandardized beta**	**Standardized beta**	**p value**
Constant	49.133		0.000
Plasma S1P	-0.003	-0.380	0.039
LDL	0.144	0.546	0.401
HDL	0.103	0.143	0.608
Cholesterine	-0.253	-1.032	0.162
Triglycerides	0.019	0.130	0.640
Culprit Vessel	-1.228	-0.126	0.483
TIMI	3.738	0.198	0.309
Fluroscopy time	-0.219	-0.337	0.528
Amount contrast media	0.010	0.121	0.827
No. of stents	-0.536	-0.058	0.752
Symptom to balloon time	0.001	0.278	0.129

S1P = Sphingosin-1-Phosphat, LDL = low-density lipoprotein, HDL = high-density lipoprotein, TIMI = Thrombolysis in Myocardial Infarction, No = number

Characteristics of included patients before IPTW

	**High (N=42)**	**Medium (N=43)**	**Low (N=42)**	**p value**
**Characteristic**				
Age	63.76 ± 13.70	62.28 ± 13.67	62.76 ± 13.53	.878
Male Gender	32 (76.2%)	31 (72.1%)	29 (69.0%)	.763
BMI	27.48 ± 4.57	28.36 ± 4.23	26.53 ± 5.23	.205
DM	9 (21.4%)	9 (20.9%)	10 (23.8%)	.943

Characteristics of included patients after IPTW

	**High (N=42)**	**Medium (N=43)**	**Low (N=43)**	**p value**
**Characteristic**				
Age	62.63 ± 13.74	63.22 ± 14.02	63.65 ± 12.77	.941
Male Gender	30 (71.4%)	30 (71.4%)	32 (74.4%)	.938
BMI	27.54 ± 4.70	27.73 ± 3.96	28.32 ± 6.35	.765
DM	9 (21.4%)	9 (21.4%)	10 (23.3%)	.973

BMI = body mass index, DM = diabetes mellitus

Supplementary Figure 1

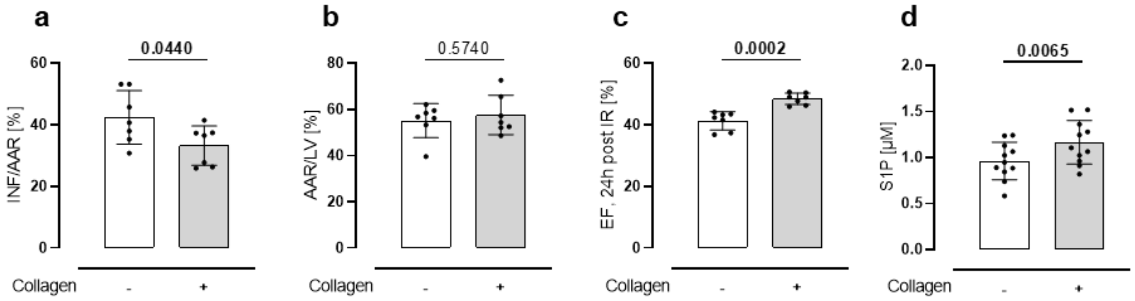

Collagen-generated SNT+ exerted cardioprotection. a,b Injection of cell-free supernatant of by collagen (10µg/ml) activated platelets (SNT+) prior AMI leads to decreased infarct size after 24 h of reperfusion as compared to supernatant of non-activated platelets (SNT-, t-test, n=7). **c** Echocardiographic assessment 24h post AMI showed improved cardiac function in SNT+ treated mice (t-test, n=7). **d** S1P content in SNT from collagen-treated human platelets is increased compared to SNT of unstimulated platelets (paired t-test, n=11). Error bars in each panel represent standard deviation.

Supplementary Figure 2

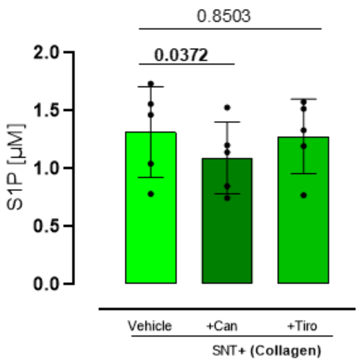

Platelet S1P release during activation by collagen (10µg/ml). This was reduced by P2Y12 inhibition (Cangrelor) but preserved during GPIIb/IIIa inhibition (Tirofiban, ANOVA-analysis, n=5). Error bars in each panel represent standard deviation.

Supplementary Figure 3

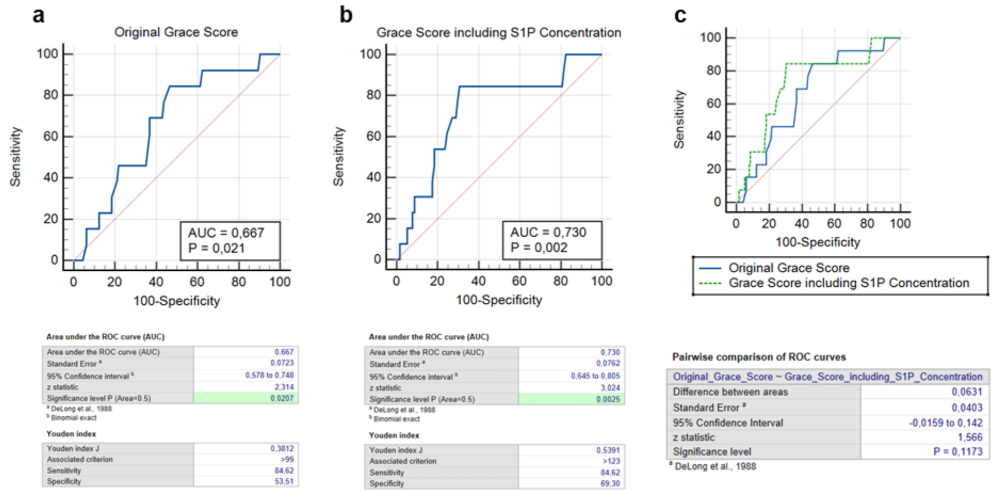

S1P improves reclassification of patients. a ROC curve of original GRACE score on 6-month mortality. **b** ROC of new GRACE score including S1P concentration on 6-month mortality. **c** Pairwise comparison of **a**+**b** ROC curves

Supplementary Figure 4

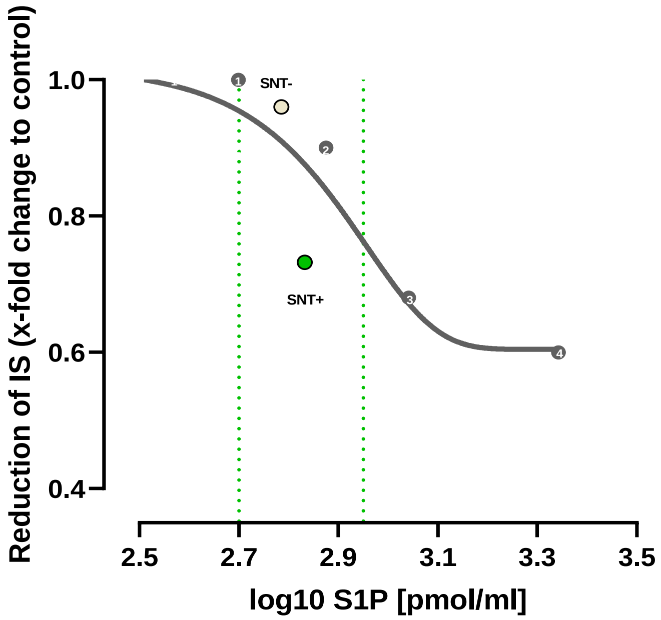

Dose-response curve between plasma S1P and reduction of infarct size. A sigmoidal dose-response curve between plasma S1P and reduction of infarct size after I/R shows a narrow slope range. The figure is based on n=6 individual data sets with a total of n=71 mice in the same model. Studies in detail from left to right: (1) untreated mice, SNT- and SNT+ treated mice from this study and mice treated with 3,8 ng/g, 19 ng/g, and 38ng/g S1P (2, 3, 4) administered intravenously 15 min prior to AMI[1].

References:

1 Theilmeier, G. *et al.* High-density lipoproteins and their constituent, sphingosine-1-phosphate, directly protect the heart against ischemia/reperfusion injury in vivo via the S1P3 lysophospholipid receptor. *Circulation* **114**, 1403-1409, doi:10.1161/circulationaha.105.607135 (2006).
