# Supplementary material for: Revealing concealed cardioprotection by platelet Mfsd2b-released S1P in human and murine myocardial infarction
Source: Nat Commun. 2023 Apr 26;14:2404. doi: 10.1038/s41467-023-38069-5 (PMC10133218; doi:10.1038/s41467-023-38069-5)
Supplement: Supplementary file 3 — Reporting Summary [file 41467_2023_38069_MOESM3_ESM.pdf]

nature portfolio

Corresponding author(s): Bodo Levkau

Last updated by author(s): Mar 31, 2023

Reporting Summary

Nature Portfolio wishes to improve the reproducibility of the work that we publish. This form provides structure for consistency and transparency in reporting. For further information on Nature Portfolio policies, see our Editorial Policies and the Editorial Policy Checklist.

Statistics

For all statistical analyses, confirm that the following items are present in the figure legend, table legend, main text, or Methods section.

n/a	Confirmed	
☐	☒	The exact sample size (*n*) for each experimental group/condition, given as a discrete number and unit of measurement
☐	☒	A statement on whether measurements were taken from distinct samples or whether the same sample was measured repeatedly
☐	☒	The statistical test(s) used AND whether they are one- or two-sided<br>*Only common tests should be described solely by name; describe more complex techniques in the Methods section.*
☒	☐	A description of all covariates tested
☐	☒	A description of any assumptions or corrections, such as tests of normality and adjustment for multiple comparisons
☐	☒	A full description of the statistical parameters including central tendency (e.g. means) or other basic estimates (e.g. regression coefficient) AND variation (e.g. standard deviation) or associated estimates of uncertainty (e.g. confidence intervals)
☒	☐	For null hypothesis testing, the test statistic (e.g. *F*, *t*, *r*) with confidence intervals, effect sizes, degrees of freedom and *P* value noted<br>*Give P values as exact values whenever suitable.*
☒	☐	For Bayesian analysis, information on the choice of priors and Markov chain Monte Carlo settings
☒	☐	For hierarchical and complex designs, identification of the appropriate level for tests and full reporting of outcomes
☐	☒	Estimates of effect sizes (e.g. Cohen's *d*, Pearson's *r*), indicating how they were calculated

Our web collection on statistics for biologists contains articles on many of the points above.

Software and code

Policy information about availability of computer code

Data collection	Analysis of infarct size: Diskus View, Technisches Büro Hilgers, Version 4.81.,<br>Echocardiographical assessment: Vevo 3.2.6., Visual Sonics, Fujifilm,<br>Analysis of flow cytometry: FlowJo v.9, BD Biosciences<br>Langendorff perfused heart: LabChart 5, ADInstruments<br>Histological pictures: LAS X, Leica<br>Cardiac magnet resonance imaging, post processing anlyses: cmr42, Circle Cardiovascular Imaging Inc., Calgary, Alberta, Canada and Extended Workspace, Philips Healthcare, Hamburg, Germany
Data analysis	Data analysis: SPSS 25, IBM; GraphPad PRISM Software 9.3.1

For manuscripts utilizing custom algorithms or software that are central to the research but not yet described in published literature, software must be made available to editors and reviewers. We strongly encourage code deposition in a community repository (e.g. GitHub). See the Nature Portfolio guidelines for submitting code & software for further information.

Data

Policy information about availability of data

All manuscripts must include a data availability statement. This statement should provide the following information, where applicable:

- Accession codes, unique identifiers, or web links for publicly available datasets
- A description of any restrictions on data availability
- For clinical datasets or third party data, please ensure that the statement adheres to our policy

The datasets generated during and/or analysed during the current study are available from the corresponding author on reasonable request.

Field-specific reporting

Please select the one below that is the best fit for your research. If you are not sure, read the appropriate sections before making your selection.

☒ Life sciences ☐ Behavioural & social sciences ☐ Ecological, evolutionary & environmental sciences

For a reference copy of the document with all sections, see nature.com/documents/nr-reporting-summary-flat.pdf

Life sciences study design

All studies must disclose on these points even when the disclosure is negative.

Sample size	As this was an explorative study, no sample size calculation was performed. However, sample size was chosen due to previous experience in murine models of cardiac ischemia/reperfusion.
Data exclusions	No data had to be excluded.
Replication	We used different experimental methods to verify our findings. Two models of murine ischemia/reperfusion were used to confirm the effect of SNT. Echocardiographic assessment confirmed our findings concerning infarct size determination.<br>The effects of Cangrelor and Tirofiban on platelet degranulation were analyzed in multiple experiments (p-selectin, ATP-release).<br>The small SD are proof of the reproducibility of our results.
Randomization	For the human data randomization was not applicable since patients were grouped according to their S1P levels. Determination of S1P levels was conducted after 12 months of follow-up.<br>For the murine experiments an inbred mouse strain was used. The mice were treated randomly.
Blinding	For the murine experiments surgery and application of SNT/medication were performed by a different person then the data analysis. The person analysing the data was blinded.<br>For the human data blinding was not applicable.

Reporting for specific materials, systems and methods

We require information from authors about some types of materials, experimental systems and methods used in many studies. Here, indicate whether each material, system or method listed is relevant to your study. If you are not sure if a list item applies to your research, read the appropriate section before selecting a response.

Materials & experimental systems

n/a	Involved in the study	
☐	☒	Antibodies
☒	☐	Eukaryotic cell lines
☒	☐	Palaeontology and archaeology
☐	☒	Animals and other organisms
☐	☒	Human research participants
☐	☒	Clinical data
☒	☐	Dual use research of concern

Methods

n/a	Involved in the study	
☒	☐	ChIP-seq
☐	☒	Flow cytometry
☒	☐	MRI-based neuroimaging

Antibodies

Antibodies used	primary antibodies:<br>rat-anti Ly6G 1:100, Abcam (ab210204), Cambrigde UK<br>rabbit anti cleaved Caspase-3 1:400, Cell signaling (9661), Massachusetts, USA<br><br>secondary antibodies:<br>ImmPRESS®- Goat Anti-Rat IgG (Mouse Adsorbed) Polymer Kit (MP-7444, Vector Labs, Burlingame, Canada) for Ly6G staining and the ImmPRESS®-AP Goat Anti-Rabbit IgG Polymer Kit (MP-7451, Vector Labs, Burlingame, Canada) for Caspase-3 staining were used.
Validation	for validation of rat-anti Ly6G, Abcam: https://www.abcam.com/ly6g-antibody-1a8-low-endotoxin-azide-free-ab210204.html<br>see references:<br>Dhanesha N et al. Cellular fibronectin promotes deep vein thrombosis in diet-induced obese mice. J Thromb Haemost 19:814-821 (2021)<br>Wen G et al. Genetic and Pharmacologic Inhibition of the Neutrophil Elastase Inhibits Experimental Atherosclerosis. J Am Heart Assoc 7:N/A (2018)<br><br>for validation of rabbit-anti cleaved Caspase 3, Cell signaling: https://www.cellsignal.com/products/primary-antibodies/cleaved-caspase-3-asp175-antibody/9661

see references:
Riera-Tur I, Schäfer T, Hornburg D, et al. Amyloid-like aggregating proteins cause lysosomal defects in neurons via gain-of-function toxicity. Life Sci Alliance. (2021)
Kolodkin-Gal D, Roitman L, Ovadya Y, et al. Senolytic elimination of Cox2-expressing senescent cells inhibits the growth of premalignant pancreatic lesions. Gut. (2022)

Animals and other organisms

Policy information about studies involving animals; ARRIVE guidelines recommended for reporting animal research

Laboratory animals	C57BL6/J, both sexes, 12-15 weeks at the beginning of the experiments<br>Edg1 CardioCre+/- mice (B6;Cg-S1P1αMHCCre+ /B6;Cg-S1P1αMHCCre-), both sexes, 12-15 weeks at the beginning of the experiments<br>C57BL/6N-Mfsd2btm1a(KOMP)Wtsi/Wtsi, both sexes, 12-15 weeks at the beginning of the experiments
Wild animals	This study did not involve wild animals.
Field-collected samples	This study did not involve field-collected samples.
Ethics oversight	All mice experiments were approved by Landesamt für Natur, Umwelt und Verbraucherschutz Nordrhein-Westfalen (LANUV NRW) and in accordance with the European Convention for the Protection of Vertebrate Animals used for Experimental and other Scientific Purposes (Council of Europe Treaty Series No. 123) and 2010/63/EU.

Note that full information on the approval of the study protocol must also be provided in the manuscript.

Human research participants

Policy information about studies involving human research participants

Population characteristics	A detailed description of covariate-relevant patient characteristics can be found in suppl. Table 1 and 2.
Recruitment	We conducted a hypothesis generating, prospective, monocentric, time-series, translational analysis in 127 patients with ST-elevation myocardial infarction (STEMI). An all-comers design was applied. Inclusion criteria were age ≥ 18 years, occurrence of one of the above-mentioned events and written informed consent. Exclusion criteria were malignant comorbidities and coagulopathies. Written informed consent was obtained from all participants.
Ethics oversight	The study conformed to the Declaration of Helsinki and was approved by the University of Düsseldorf Ethics Committee.

Note that full information on the approval of the study protocol must also be provided in the manuscript.

Clinical data

Policy information about clinical studies

All manuscripts should comply with the ICMJE guidelines for publication of clinical research and a completed CONSORT checklist must be included with all submissions.

Clinical trial registration	NCT03539133
Study protocol	The full trial protocol is available from the correspondig author on request.
Data collection	Blood sampling was conducted during ischemia as well as 12 hours after intervention. To assess infarct size, gadolinium-based contrast agent (Gadovist, Bayer Healthcare, Berlin, Germany 0.2 mmol/kg) was used to detect late gadolinium enhancement in cardiac magnet resonance imaging 6 months after STEMI during clinical follow-up. Cardiovascular death was assessed during 12 months follow-up.
Outcomes	n/a

Flow Cytometry

Plots

Confirm that:

- [ ] The axis labels state the marker and fluorochrome used (e.g. CD4-FITC).
- [ ] The axis scales are clearly visible. Include numbers along axes only for bottom left plot of group (a 'group' is an analysis of identical markers).
- [ ] All plots are contour plots with outliers or pseudocolor plots.
- [ ] A numerical value for number of cells or percentage (with statistics) is provided.

Methodology

Sample preparation	Citrate-anticoagulated whole blood was centrifuged at 300g for 10 minutes to gain PRP. 100 µl of PRP were incubated for 6 minutes at 37°C. After another 6 minutes of stimulation with ADP (5 µM) at 37°C, the following antibodies were added and incubated for 30 minutes at 37°C: CD41/61-APC-Cy7 (Miltenyi Biotec, Bergisch Gladbach, Germany), CD42b-Pe-Cy7 (invitrogen, Massachusetts, USA) and CD62P-BV421 (BD Biosciences, Franklin Lakes, USA). Before measurement with BD FacsVerse® (BD Biosciences, Franklin Lakes, USA), the solution was diluted 1:100 in phosphate buffered saline (PBS).
Instrument	BD FacsVerse® (BD Biosciences, Franklin Lakes, USA)
Software	FlowJo v.9, BD Biosciences
Cell population abundance	Cells of interest were selected via FSC/SSC (size) and gating of CD41/61 positive cells only. Purity of samples was > 98%.
Gating strategy	After sorting based on size of single cells (FSC-A/FSC-H), CD41/61 positive cells were selected. Within these, we determined CD62P positive cells. Boundaries were adapted for each sample and individually based on an unstained control sample (approx. 10^3 flourescence intensity).

☐ Tick this box to confirm that a figure exemplifying the gating strategy is provided in the Supplementary Information.
